# Yellow Fever Virus Surveillance in *Callithrix* spp. Marmosets during Epizootic Outbreak, Brazil, 2024–2025

**DOI:** 10.3201/eid3206.251388

**Published:** 2026-06

**Authors:** Márcio Junio Lima Siconelli, Jéssica Caroline de Almeida Dias, Eduardo Ferreira Machado, Mariana Sequetin Cunha, Natália Coelho Couto de Azevedo Fernandes, Juliana Mariotti Guerra, Luana Bonon, Alline Borges Salomão, Karin Werther, Karina Paes Bürger, Adolorata Aparecida Bianco Carvalho, Daniel Marques, Benedito Antonio Lopes da Fonseca

**Affiliations:** Unidade de Vigilância de Zoonoses da Divisão de Vigilância Ambiental em Saúde, Secretaria Municipal da Saúde, Ribeirão Preto, Brazil (M.J.L. Siconelli); Faculdade de Medicina de Ribeirão Preto, Universidade de São Paulo, Ribeirão Preto (M.J.L. Siconelli, J.C. de Almeida Dias, B.A. Lopes da Fonseca); Centro de Patologia, Instituto Adolfo Lutz de São Paulo, Secretaria Estadual da Saúde de São Paulo, São Paulo, Brazil (E.F. Machado, N.C.C.A. Fernandes); Centro de Virologia, Instituto Adolfo Lutz de São Paulo, Secretaria Estadual da Saúde de São Paulo, São Paulo (M.S. Cunha); Universidade de São Paulo Departamento de Patologia da Faculdade de Medicina Veterinária e Zootecnia, São Paulo (J.M. Guerra); Programa de Residência em Área Profissional da Saúde, Medicina Veterinária e Saúde, do Departamento de Patologia, Reprodução e Saúde Única da Faculdade de Ciências Agrárias e Veterinárias, Universidade Estadual Paulista, Jaboticabal, Brazil (L. Bonon, A.B. Salomão, K. Werther, K.P. Bürger, A.A.B. Carvalho); Grupo de Vigilância Epidemiológica, Centro de Vigilância Epidemiológica “Prof. Alexandre Vranjac,” Secretaria Estadual da Saúde de São Paulo, São Paulo (D. Marques)

**Keywords:** Yellow fever, yellow fever virus, viruses, zoonoses, vector-borne infections, nonhuman primates, epizootic, marmosets, high viral load, Councilman–Rocha Lima bodies, amplifier host, Brazil

## Abstract

In 2023, a new yellow fever virus (YFV) lineage was introduced in São Paulo state, Brazil. During July 2024–June 2025, nine *Callithrix penicillata* marmosets tested YFV–positive, showing high viral loads and characteristic organ lesions. Those results highlight the need to include these animals in multispecies surveillance strategies for early YFV detection.

Yellow fever virus (YFV; *Orthoflavivirus flavi*), is an arbovirus of the Flaviviridae family transmitted by *Aedes* spp. mosquitoes in the urban cycle and *Haemagogus*/*Sabethes* spp. mosquitoes in the sylvatic cycle (*1*,*2*). Nonhuman primates (NHPs) are key sentinel animals for early YFV detection and a core component of the Brazilian Yellow Fever Surveillance Program (*3*). However, YFV pathogenicity varies among species, and the role of Brazil’s diverse NHP populations in continued YFV circulation remains poorly understood (*4*). *Alouatta* spp. howler monkeys are the most susceptible to YFV infection (*5*–*7*). Infected NHPs develop clinical and pathologic manifestations similar to those in humans (*4*,*8*,*9*), who exhibit high viral loads and pronounced liver damage, including areas of necrosis and apoptosis, Councilman–Rocha Lima (CRL) bodies, steatosis, and inflammatory infiltrates (*10*,*11*).

During 2016–2018, Brazil experienced its worst wild yellow fever outbreak in 70 years, in which most human and NHP cases occurred in the southeastern region (*1*,*2*). In that outbreak, most infected NHPs were *Callithrix* spp. marmosets, revealing 2 infection patterns: one with a high viral load and characteristic histopathologic hepatic lesions and another with low viral loads, absence of clinical disease, and minimal or absent hepatic lesions (*4*,*12*).

In 2023, a new YFV lineage emerged in eastern São Paulo state (*13*), then spread throughout most of the state. The first confirmed NHP deaths attributed to YFV in the region of Ribeirão Preto city occurred in late December 2024. During the following months, the outbreak intensified, and more confirmed NHP infections and 1 human case occurred in the surrounding municipalities (Figure 1). We investigated clinical and laboratory findings of YFV infection among NHPs during that epizootic outbreak.

**Figure 1:**
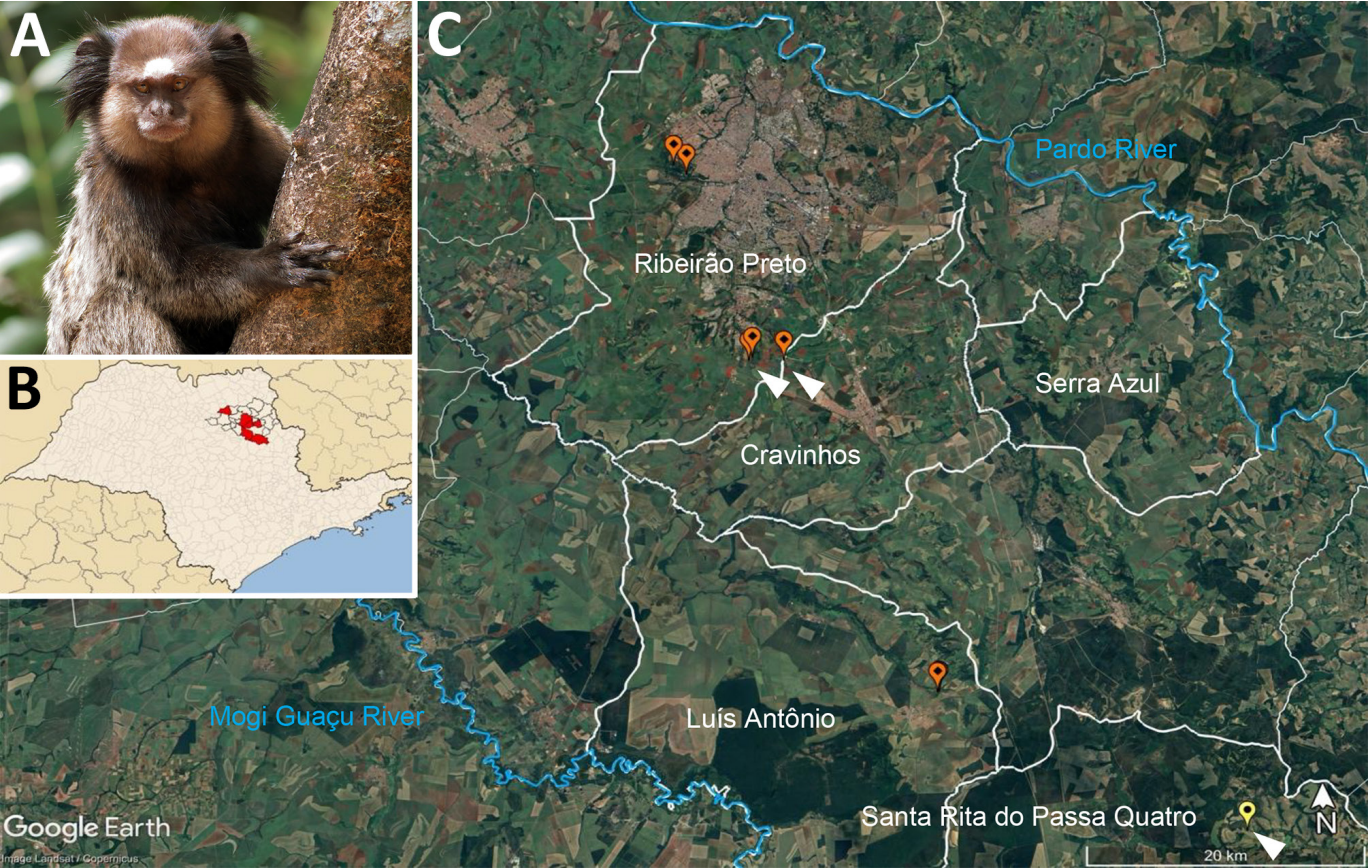
Location and example species for yellow fever virus (YFV) surveillance in *Callithrix* spp. marmosets during epizootic outbreak, Brazil, 2024–2025. A) *C. penicillata* marmoset. Source: Miguelrangeljr, CC BY-SA 3.0, via Wikimedia Commons, https://creativecommons.org/licenses/by-sa/3.0. B) São Paulo state map with dark lines highlighting the municipalities of the regional Epidemiologic Surveillance Group; red indicates areas affected by yellow fever. Map created by Raphael Lorenzeto de Abreu on WikiMedia (https://commons.wikimedia.org/w/index.php), modified by the authors and used according to permissions. C) Partial aerial view of the Ribeirão Preto region. White outlines represent borders of municipalities where YFV was detected during July 2024–June 2025. Orange dots indicate locations where YFV-positive *C. penicillata* marmosets were found; yellow dot indicates confirmed human yellow fever case; white arrowheads indicate areas without *Alouatta* spp. howler monkeys. Map from Google Earth, 2025 (https://earth.google.com).

## The Study

During July 2024–June 2025, the regional Epidemiologic Surveillance Group reported 233 NHP deaths, of which 79% (184/233) were *Callithrix penicillata* black-tufted marmosets, 18% (42/233) *Alouatta caraya* black howler monkeys, 2.15% (5/233) *Sapajus nigritus* black capuchin monkeys, and 0.85% (2/233) *Callicebus nigrifrons* black fronted titi monkeys. Among the 233 NHPs, we necropsied and collected biologic samples from 154 (66.1%), 88.9% (n = 137) of which were Callitrichid marmosets.

We confirmed YFV infection in a total of 54 NHPs: 22 by laboratory testing on organ samples collected during necropsy and 32 by epidemiologic criteria. We could not obtain samples from the 32 NHPs identified by epidemiologic criteria because the carcasses were in an advanced state of decomposition. However, we included those animals because they were found in the same epidemiologic context and area in which YFV was detected in other animals.

**Figure 2:**
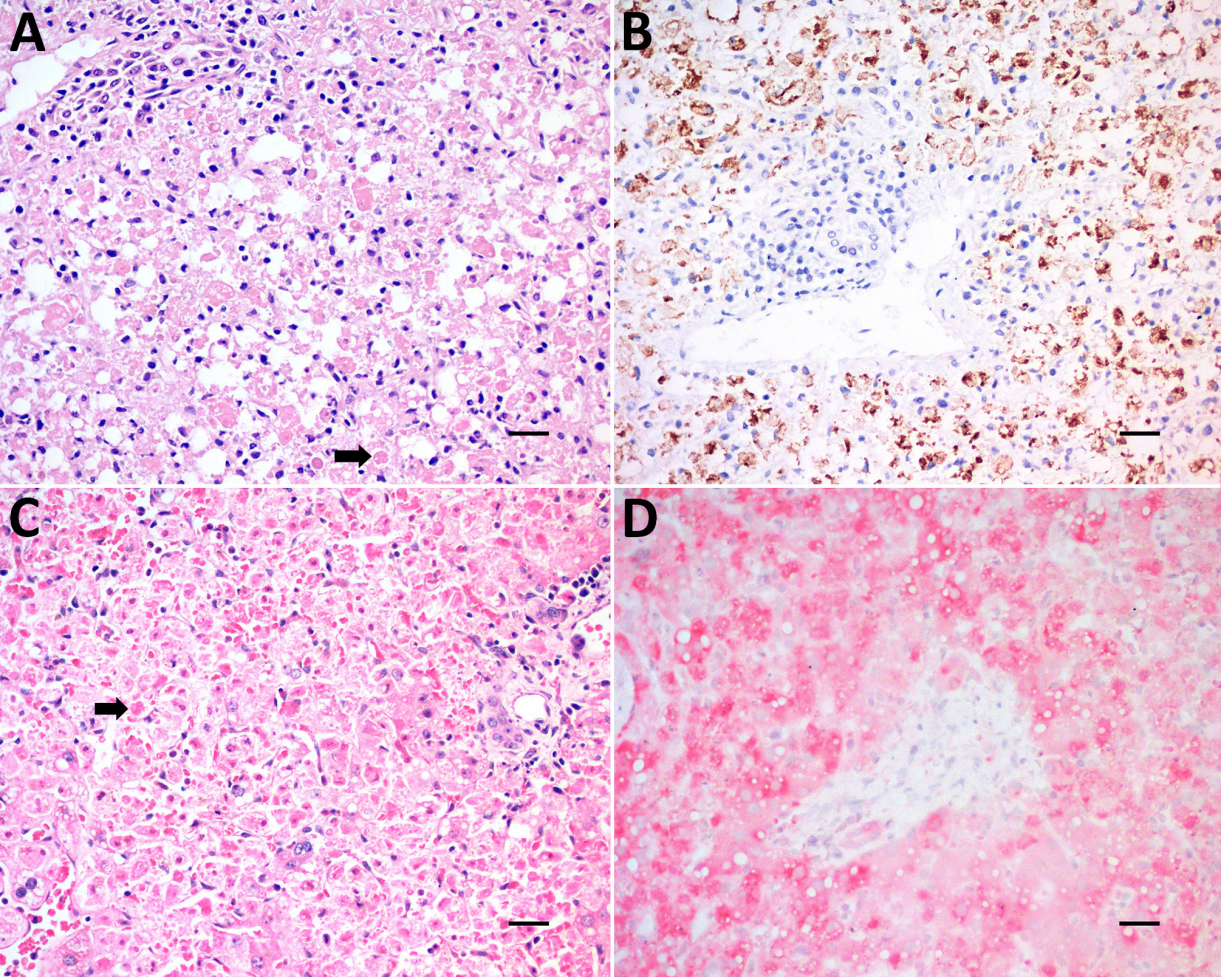
Laboratory analysis of liver samples from nonhuman primates collected for yellow fever virus (YFV) surveillance in *Callithrix* spp. marmosets during epizootic outbreak, Brazil, 2024–2025. A, B) Samples from *Callicebus nigrifrons* black fronted titi monkeys; C, D) samples from *Alouatta caraya* black howler monkeys. A) Hematoxylin and eosin stain of liver showing acute and severe hepatic damage characterized by diffusely individual cellular apoptosis and necrosis with Councilman–Rocha Lima bodies (arrow). B) Immunohistochemistry stain of hepatocytes; brown stain indicates cells positive for YFV antigen. C) Immunohistochemistry stain showing acute and severe hepatic damage characterized by individual cellular apoptosis and necrosis with Councilman–Rocha Lima bodies (arrow); brown indicates hepatocytes positive by YFV antigen. D) Immunohistochemistry stain of hepatocytes positive for YFV antigen (red). Scale bars indicate 20 µm.

Among 22 NHPs with laboratory-confirmed YFV infection, most were *A. caraya* monkeys (50%; 11/22), followed by *C. penicillata* (40.9%; 9/22) marmosets and *C. nigrifrons* (9.1%; 2/22) monkeys. As expected, samples from *Alouatta* and *Callicebus* spp. NHPs had low cycle threshold (Ct) values and typical organ lesions, including extensive hepatocellular necrosis, CRL bodies, hepatic steatosis, varying degrees of inflammatory infiltrates, and YFV antigens detectable by immunohistochemistry (IHC) (Figure 2). The YFV-positive marmosets exhibited a consistent pattern: low Ct values (Ct 11.14–20.08), typical histopathologic lesions, and detectable YFV antigens. One marmoset, stored frozen for a month before necropsy, showed a higher Ct value of 28.9. 

**Figure 3:**
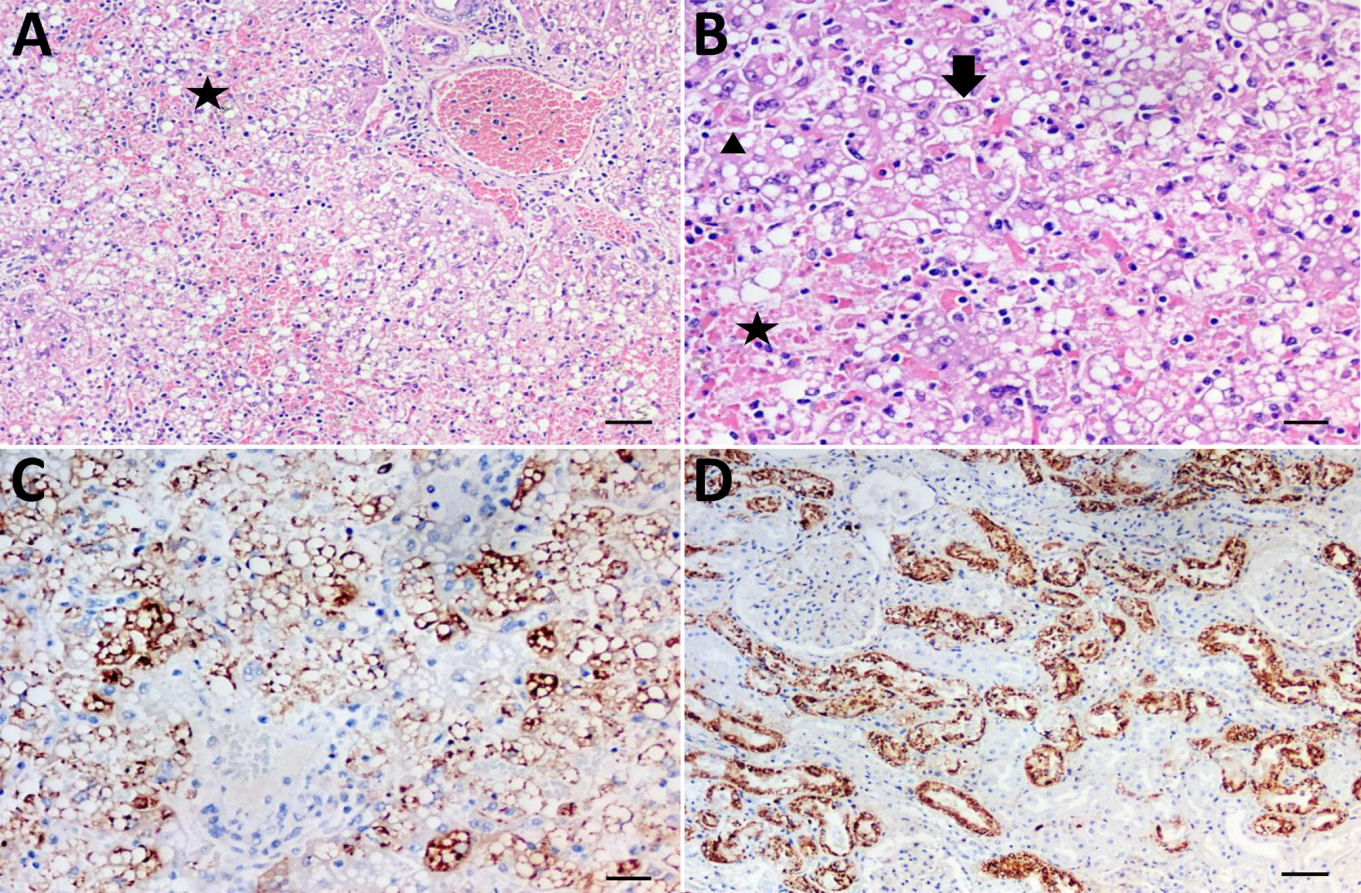
Laboratory analysis of organ samples collected from *Callithrix penicillata* marmosets with severe hepatic lesions due to yellow fever during epizootic yellow fever outbreak, Brazil, 2024–2025. A) Hematoxylin and eosin stain of liver showing acute and severe hepatic damage characterized by diffuse macrovesicular steatosis and focal hemorrhage (star). Original magnification ×10. B) Hematoxylin and eosin stain of liver showing acute and severe hepatic damage with macrovesicular steatosis (black arrowhead), focal hemorrhage (star), and a Councilman–Rocha Lima body (elongated arrow). Original magnification ×20. C) Immunohistochemisty stain showing hepatocytes positive for yellow fever virus antigen (brown staining). Original magnification ×20. D) Immunohistochemistry stain of renal tubular epithelial cells; brown stained cells are positive for yellow fever virus antigen. Original magnification ×10.

All 9 *C. penicillata* marmosets were YFV-positive by IHC (Figure 3); 2 (22%) showed advanced autolysis, which precluded evaluation of histopathologic features, but we did detect CRL bodies in 1 of those 2 marmosets. Histopathologic findings in the other 7 animals showed all had hepatic necrosis, 3 (42%) with multifocal panlobular distribution and 4 (57%) with widespread panlobular involvement. Necrosis severity ranged from moderate in 2 (29%) to marked in the other 5 (71%) animals. All 7 animals had CRL bodies, and 3 (43%) had inflammatory infiltrates, consisting of mixed cellular contents in 1 (33.3%) and mononuclear cells in the other 2 (66.7%); we observed multifocal distribution and moderate severity in 2 (66.7%) cases and discrete distribution in 1 (33.3%). We identified hemorrhage in 4 (57%) of those animals, characterized by diffuse distribution in most (75%; 3/4); 3 (75%) exhibited moderate severity and 1 (25%) marked severity. We observed steatosis in 6 (86%) animals and panlobular macrovesicular and microvesicular patterns of moderate severity in 2 (34%) and marked severity in 4 (66%).

During the 2016–2018 outbreak, 7 *Callithrix* spp. marmosets were YFV-positive, 5 of which were from Ribeirão Preto and neighboring cities, but none had hepatic lesions or YFV antigens detected (*14*). In 2020, an urban *C. penicillata* marmoset from midwestern Brazil showed the same pattern, YFV-positive by quantitative reverse transcription PCR but no liver lesions nor YFV antigen detection (*15*).

The IHC and molecular findings of this study, representing the data from the 2024–2025 epizootic outbreak in São Paulo state demonstrate a consistent pattern of severe acute hepatitis in neotropical primates infected with YFV. All 22 confirmed NHP cases revealed characteristic hepatic lesions, with hepatocellular necrosis in all evaluated specimens, predominantly showing diffuse and multifocal to coalescing patterns. Those findings corroborate previous reports describing panlobular necrosis as a hallmark of fatal YFV infection in primates (*4*). 

We identified CRL bodies, characteristic of YFV lesions, in 80% (18/22) of cases, and noted a much higher frequency in *A. caraya* and *C. nigrifrons* monkeys and *C. penicillata* marmosets. Even in specimens with marked autolysis (typically observed 1–3 days after death) CRL bodies remained detectable in 3 NHPs, reinforcing the strong association of CRL bodies with YFV infections, even under suboptimal tissue preservation conditions. Inflammatory infiltrates were predominantly mixed (polymorphonuclear and mononuclear) or mononuclear, with panlobular or periportal and portal distribution. We observed macrovesicular, microvesicular, or mixed steatosis with diffuse or multifocal distribution and consistent panlobular zonation. Severity was marked to moderate in 72.2% (13/18) of affected animals.

The high prevalence and severity of lesions combined with limited inflammatory response, despite extensive necrosis and steatosis, might indicate a YFV-induced metabolic dysfunction and support YFV as the main mechanism of liver injury. The histopathologic data we present need further investigation because they point to a possible association with the new YFV lineage circulating in southern Brazil. A relevant finding that supports that hypothesis is IHC confirmation of active YFV infection in 100% of cases, including all analyzed *C. penicillata* marmoset specimens. That contrasts with prior observations during 2017–2018, when *Callithrix* spp. marmosets often exhibited lower viral loads and milder hepatic lesions (*4*,*14*,*15*). In the NHPs evaluated here, marmosets had severe liver damage comparable to that seen in howler monkeys, with extensive necrosis and unequivocal IHC positivity. The severe lesions observed in *Callithrix* spp. marmosets underscore this primate’s relevance in NHP-based YFV surveillance, particularly in areas where *Alouatta* spp. monkey populations have been greatly reduced.

## Conclusions

Our results confirm the classic histopathologic profile of YFV infection in neotropical primates, with striking severity in *C. penicillata* marmosets during an outbreak in an area with reduced *Alouatta* spp. monkey populations. The combination of hepatocellular necrosis, CRL bodies, steatosis, and 100% IHC positivity provides robust evidence for YFV pathogenicity across multiple primate species and reinforces the need for continuous surveillance in NHPs, placing *Callithrix* genus NHPs under the public health spotlight in this and in future epidemics. Its ability to adapt to urbanized environments, its proximity to humans, and the high *Ae. aegypti* mosquito population raise concerns for yellow fever reurbanization. 

Our findings underscore the need for establishing a new NHP surveillance system based on *Callithrix* spp. marmosets in areas where *Alouatta* spp. monkey populations have been reduced or extinguished. Therefore, local zoonotic surveillance services in high-risk areas must be strengthened to detect early YFV circulation in the face of current NHP deaths, especially in view of a possible change in the yellow fever epidemiologic pattern.
